# *Stauntonia hexaphylla* leaf extract (YRA-1909) suppresses inflammation by modulating Akt/NF-κB signaling in lipopolysaccharide-activated peritoneal macrophages and rodent models of inflammation

**DOI:** 10.29219/fnr.v65.7666

**Published:** 2021-10-25

**Authors:** Jaeyong Kim, Gyuok Lee, Huwon Kang, Ji-Seok Yoo, Yongnam Lee, Hak-sung Lee, Chul-yung Choi

**Affiliations:** 1 Jeonnam Bioindustry Foundation, Jeonnam Institute of Natural Resources Research (JINR), Jeollanamdo, Republic of Korea; 2 Rexpharmtech. Co., Ltd., Yongin, Seoul, Republic of Korea; 3 Department of Biomedical Science College of Natural Science, Chosun University, Gwangju, Republic of Korea

**Keywords:** Stauntonia hexaphylla, YRA-1909, anti-inflammatory, NF-κB, Akt, neochlorogenic acid, cryptochlorogenic acid, chlorogenic acid

## Abstract

**Background:**

Inflammation is emerging as a key contributor to many vascular diseases and furthermore plays a major role in autoimmune diseases, arthritis, allergic reactions, and cancer. Lipopolysaccharide (LPS), which is a component constituting the outer membrane of Gram-negative bacteria, is commonly used for an inflammatory stimuli to mimic inflammatory diseases. Nuclear factor-kappa B (NF-κB) is a transcription factor and regulates gene expression particularly related to the inflammatory process. *Stauntonia hexaphylla* (Lardizabalaceae) is widely used as a traditional herbal medicine for rheumatism and osteoporosis and as an analgesic, sedative, and diuretic in Korea, Japan, and China.

**Objective:**

The purpose of this study was to investigate the anti-inflammatory activity of YRA-1909, the leaf aqueous extract of *Stauntonia hexaphylla* using LPS-activated rat peritoneal macrophages and rodent inflammation models.

**Results:**

YRA-1909 inhibited the LPS-induced nitric oxide (NO) and proinflammatory cytokine production in rat peritoneal macrophages without causing cytotoxicity and reduced inducible NO synthase and prostaglandin E_2_ levels without affecting the cyclooxygenase-2 expression. YRA-1909 also prevented the LPS-stimulated Akt and NF-κB phosphorylation and reduced the carrageenan-induced hind paw edema, xylene-induced ear edema, acetic acid-induced vascular permeation, and cotton pellet-induced granuloma formation in a dose-dependent manner in mice and rats.

**Conclusions:**

*S. hexaphylla* leaf extract YRA-1909 had anti-inflammatory activity *in vitro* and *in vivo* that involves modulation of Akt/NF-κB signaling. Thus, YRA-1909 is safe and effective for the treatment of inflammation.

## Popular scientific summary

- YRA-1909 inhibited the LPS-induced NO and proinflammatory cytokine production in rat peritoneal macrophages without causing cytotoxicity and reduced iNOS and PGE_2_ levels without affecting the COX-2 expression.
- YRA-1909 prevented the LPS-stimulated Akt and NF-κB phosphorylation and dose-dependently reduced carrageenan-induced hind paw edema, xylene-induced ear edema, acetic acid-induced vascular permeation, and cotton pellet-induced granuloma formation.

Inflammation is a protective biological response to harmful stimuli such as injury, infection, and irritants (1, 2). Uncontrolled and chronic inflammations can lead to ailments such as Alzheimer’s disease; autoimmune, cardiovascular, and inflammatory bowel diseases; rheumatoid arthritis; allergy; asthma; diabetes; and cancer (2, 3).

Macrophages play an important role in inflammation by producing nitric oxide (NO) and prostaglandin (PG)E_2_, which are generated by activated inducible NO synthase (iNOS) and cyclooxygenase (COX)-2 and cytokines such as interleukin (IL)-1β, interleukin (IL)-6, and tumor necrosis factor (TNF)-α in response to various stimuli (4, 5). Lipopolysaccharide (LPS)-stimulated macrophages disrupt the intracellular reduction–oxidation balance leading to oxidative stress, which is generally accompanied by reactive oxygen species-mediated cellular damage (6).

Nuclear factor-kappa B (NF-κB) is among the most important transcription factors regulating the expression of inflammatory mediators (7). It mainly exists as a heterodimer composed of the Rel family proteins p50 and p65. Under normal conditions, NF-κB is localized in the cytoplasm where it is bound by the inhibitor of (I)κB proteins (IκBα, IκBβ, and IκBε). However, upon exposure to inflammatory stimuli such as LPS, IκB is phosphorylated by IκB kinase (IKK) and then degraded by the proteasome (8). NF-κB is released and translocates to the nucleus, where it activates the transcription of multiple genes via a *cis*-acting κB element. NF-κB also interacts with multiple upstream factors to regulate LPS-induced, macrophage-mediated inflammation involving phosphoinositide 3-kinase (PI3K)/Akt (9).

Synthetic non-steroidal anti-inflammatory drugs (NSAIDs) are widely used for the treatment of inflammatory diseases (10). However, currently available NSAIDs cause serious side effects such as gastric lesions, renal damage, bronchospasm, and cardiac abnormalities (11). Therapeutic strategies that modulate the NF-κB signaling pathway are considered as a promising alternative to NSAIDs for the treatment of inflammation.

Plants are an important source of pharmacologically active substances (12, 13). Natural plant extracts contain many phytochemicals and have been used to treat inflammatory diseases (14, 15), and recent studies have demonstrated the anti-inflammatory activity of plant extracts (16–18). Thus, plant-derived medicines are of great interest for the treatment of various medical conditions, including inflammation-related ailments owing to their diverse bioactive substances and relatively low toxicity (15, 19).

**Fig. 1:**
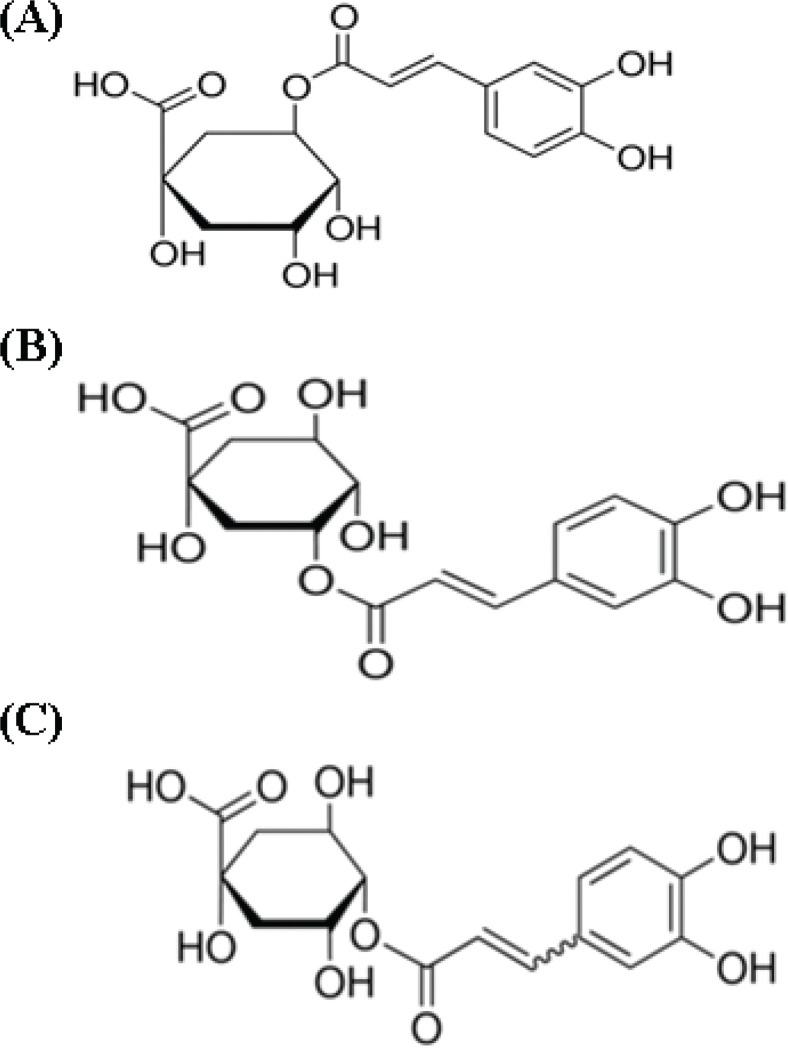
Structures of the major compounds in YRA-1909. (a) Neochlorogenic acid, (b) chlorogenic acid, and (c) cryptochlorogenic acid.

*Stauntonia hexaphylla* is an evergreen climber with palmate leaves and small, bell-shaped flowers that produce edible fruit. It is widely distributed in Korea, Japan, and China and has been used as an analgesic, sedative, and diuretic in folk medicine (20, 21). It also exhibits anti-osteoporotic (22), antioxidant, and antidiabetic activities (23). The main chemical constituents are flavonoids, triterpenoids, glycosides, and saponins (21, 24). The *S. hexaphylla* leaf extract YRA-1909 has been shown to exert antirheumatic effects (25) and is in phase 2 clinical trials in Korea for the safety and efficacy evaluations in patients with rheumatoid arthritis (ClinicalTrials.gov identifier NCT03275025). Chlorogenic acid (CGA), neochlorogenic acid (NCGA), and cryptochlorogenic acid (CCGA) have been identified as the active ingredients in *S. hexaphylla* (23, 26) and YRA-1909 (Fig. 1); CGA and NCGA have been reported to exhibit antioxidant (27), anticancer (28), antiviral (29), anti-inflammatory (30, 31), anticoagulant, and antithrombotic (32) activities.

The present study investigated the anti-inflammatory properties of YRA-1909 *in vivo*. Our results demonstrate that YRA-1909 inhibits the expression, secretion, and activity of NO, proinflammatory cytokines, PGE_2_, iNOS, and COX-2 in LPS-stimulated rat peritoneal macrophages by suppressing Akt-mediated NF-κB activity.

## Materials and methods

### Material and chemicals

*S. hexaphylla* leaves were collected from cultivated fields in accordance with Good Agricultural Practice guidelines in Jangheung-gun, Jeollanam-do, Korea. YRA-1909 was prepared by extracting *S. hexaphylla* leaves with distilled water followed by column chromatography purification, which was performed by KGCYebon (Chungju, Korea), according to the protocol recommended by the International Conference on Harmonization and Good Manufacturing Practice. The CGA, NCGA, and CCGA contents of YRA-1909 (batch no. YR-1001) were 4.74, 1.10, and 3.49 mg/g, respectively, as determined by high-performance liquid chromatography (Fig. 1). Dulbecco’s modified Eagle’s medium (DMEM) and fetal bovine serum (FBS) were purchased from Gibco (Grand Island, NY, USA). The Enzyme-Linked Immunosorbent Assay (ELISA) Kits for PGE_2_ and COX-2 inhibitor were obtained from Cayman Chemicals (San Diego, CA, USA). TNF-α, IL-1, and IL-6 were purchased from R&D Systems (Minneapolis, MN, USA). Mouse (IκB, phosphorylated [p-]IκBα, NF-κB, Akt, β-actin, and proliferating cell nuclear antigen [PCNA]) and rat (COX-2, iNOS, and NF-κB) antibodies were purchased from Cell Signaling Technology (Danvers, MA, USA). Other chemicals were obtained from Sigma-Aldrich (St. Louis, MO, USA).

### Preparation of peritoneal macrophages and cell culture

Peritoneal macrophages were collected from rats 3 days after the intraperitoneal injection of 10 mL of 4% (w/v) fluid thioglycollate medium as previously reported (33). The harvested cells were washed using Hanks’ Balanced Salt Solution (Gibco) and cultured in DMEM supplemented with 100 U/mL penicillin, 100 µg/mL streptomycin, and 10% FBS in a flat-bottom culture plate at 37°C in an atmosphere of 5% CO_2_.

### Cell viability assay

Peritoneal macrophages (4 × 10^5^ cells/well) were cultured in 48-well plate containing DMEM supplemented with 10% FBS for 24 h. The cells were pretreated with YRA-1909 (50, 100, and 200 μg/mL) for 18 h, and 3-(4, 5-dimethylthiazol-2-yl)-2,5-diphenyltetrazolium bromide solution (50 μg /well) was added for 4 h. The medium was replaced with 100 μL of dimethyl sulfoxide followed by incubation for 30 min. Absorbance at 570 nm was measured using a microplate reader.

### Measurement of NO and PGE2 production

Peritoneal macrophages (5 × 10^5^ cells/mL) seeded in 48-well plates were pre-incubated for 2 h. YRA-1909 (50, 100, and 200 μg/ml) was added to the cells along with LPS (1 μg/mL) at 37°C for 18 h. The NO level was determined by detecting nitrite, the stable product of the reaction between NO and molecular oxygen in the culture supernatant using Griess reagent. PGE_2_ levels were measured with an ELISA kit, according to the manufacturer’s protocol. Briefly, macrophages (1 × 10^5^ cells/well) were incubated with LPS (1 μg/mL) in the absence or presence of YRA-1909 (50, 100, and 200 µg/mL) for 18 h, and the PGE_2_ level in the supernatant was determined.

### Measurement of proinflammatory cytokine production

Peritoneal macrophage cells were plated at 2 × 10^6^ and stimulated with LPS for 18 h in the presence or absence of YRA-1909 (50, 100, and 200 µg/mL). The levels of proinflammatory cytokines (IL-1β, IL-6, and TNF-α) in the supernatant were measured using mouse ELISA kits.

### Western blot analysis

Western blot analysis was performed by lysing the cells in radioimmunoprecipitation assay buffer composed of 25 mM Tris–HCl (pH 7.4), 150 mM NaCl, 1% Nonidet P-40, 1% sodium deoxycholate, and 0.1% sodium dodecyl sulfate (SDS) and containing a protease inhibitor mixture. The protein concentration was determined using the Bradford assay; the absorbance at 595 nm was measured on a microplate reader. An equal amount of protein for each sample was resolved by 8–10% SDS polyacrylamide gel electrophoresis and then electrophoretically transferred to a polyvinylidene difluoride membrane (Roche, Mannheim, Germany) that was blocked with 5% skim milk and incubated overnight at 4°C with primary antibodies against COX-2 (SC-1747; Santa Cruz Biotechnology, Santa Cruz, CA, USA), iNOS (#39898, Cell Signaling Technology, Danvers, MA, USA), NF-κB (#6956, Cell Signaling Technology), IκBα (#9242, Cell Signaling Technology), Akt (#9272, Cell Signaling Technology), p-Akt (#9271, Cell Signaling Technology), β-actin (#4967, Cell Signaling Technology, Danvers), and PCNA (SC-7907, Santa Cruz Biotechnology) used at 1:1000 dilution. The membrane was treated with horseradish peroxidase-conjugated secondary antibody for 1 h at 4°C, and proteins were detected by enhanced chemiluminescence (Animal Genetics, Tallahassee, FL, USA).

### Measurement of COX-2 inhibitor activity

COX-2 inhibitor activity was measured using a fluorescent activity assay kit (Cayman Chemicals, San Diego, CA, USA), according to the manufacturer’s specifications. Briefly, peritoneal macrophages (1 × 10^5^ cells/well) were incubated with YRA-1909 (50, 100, or 200 µg/mL), followed by LPS (100 ng/mL) for 18 h, and the COX-2 enzymatic activity in the supernatant was evaluated.

### Experimental animals

ICR mice weighing 18–22 g and Sprague-Dawley rats weighing 180–220 g were purchased from Samtako Bio (Seoul, Korea). The animals were maintained on a 12:12-h light/dark cycle under a controlled temperature (22 ± 2°C) and humidity (50 ± 10%) and had free access to a standard diet and water. The animal study was approved by the Institutional Animal Care and Use Committee (IACUC) of Jeollanamdo Institute of Natural Resources Research (approval no. JINR1804), and all animal experiments were conducted in accordance with IACUC guidelines.

### Xylene-induced ear edema in mice

The xylene-induced ear edema test was performed as previously described (34), with some modifications. Briefly, mice were intragastrically administered YRA-1909 (50–200 mg/kg body weight [b.w.]) or the positive control celecoxib (4-[5-(4-methylphenyl)-3-(trifluromethyl)-1H-pyrazol-1-yl] benzenesulfonamide; 60 mg/kg b.w.) 1 h before the induction of ear edema by the topical application of 30 μL xylene to the inner and outer surfaces of the right ear; the untreated left ear served as the control. Mice were sacrificed by cervical dislocation 1 h after xylene application. Ear biopsies 8.0 mm in diameter were obtained and weighed, and the extent of ear edema was evaluated by the weight difference between the right and the left ear biopsies from the same animal.

### Carrageenan-induced rat paw edema

Carrageenan-induced rat hind paw edema was evaluated as previously described (35). Briefly, rats were intragastrically administered test sample (50–200 mg/kg YRA-1909) or the positive control substance (60 mg/kg celecoxib) 1 h before the carrageenan injection. Edema was induced by subcutaneous injection of 0.1 mL carrageenan (1% in saline) into the right hind paw of each rat. The paw volume was determined before and 1, 2, 3, 4, and 5 h after the carrageenan injection using a plethysmometer (Ugo Basile, Comerio, Italy). The percentage of edema inhibition was calculated using the formula ([C_t_ − C_0_]/C_0_) × 100, where C_0_ and C_t_ are the average percentages for the control and test groups, respectively.

### Acetic acid-induced vascular permeability in mice

The experiment was carried out as previously described (18). Briefly, mice were intragastrically administered YRA-1909 or celecoxib, and 1 h later, each mouse was intravenously injected with 0.5% Evans Blue solution at 0.1 mL/10 g b.w., followed by intraperitoneal injection of 0.6% acetic acid at 0.1 mL/10 g b.w. Mice were sacrificed by cervical dislocation 30 min after acetic acid injection, and the peritoneal cavity of each animal was washed three times with a total of 10 mL of saline. Saline washes from the same animal were combined and centrifuged for 10 min at 1,000 rpm in a tabletop centrifuge. The supernatant was collected, and the absorbance at 590 nm was measured with a spectrophotometer. The amount of Evans Blue extruded into the peritoneal cavity was estimated from a standard curve.

### Cotton pellet-induced granuloma formation in rats

The cotton-pellet implantation experiment was performed in rats as previously described (36), with some modifications. Briefly, each rat was anesthetized with ether, and a 40-mg sterile cotton pellet was implanted into the groin region. The rat was intragastrically administered YRA-1909 (50–200 mg/kg b.w.) or celecoxib (60 mg/kg b.w.) or vehicle once daily for 7 consecutive days; they were then sacrificed by decapitation on day 8, and the cotton pellets were carefully removed and cleaned of any tissue before drying in an oven at 60°C until a constant weight was obtained. Granuloma formation was evaluated based on the dry weight of the pellet.

### Statistical analysis

Results are expressed as mean ± standard deviation (SD). Comparisons between groups were performed by one-way analysis of variance. Differences between individual treatment groups were evaluated using Dunnett’s test. Statistical significance was set at *P* < 0.05. Statistical analyses were performed using Prism v.5.0 software (GraphPad, La Jolla, CA, USA).

## Results

### Effect of YRA-1909 on LPS-induced NO and PGE2 production in rat peritoneal macrophages

**Fig. 2:**
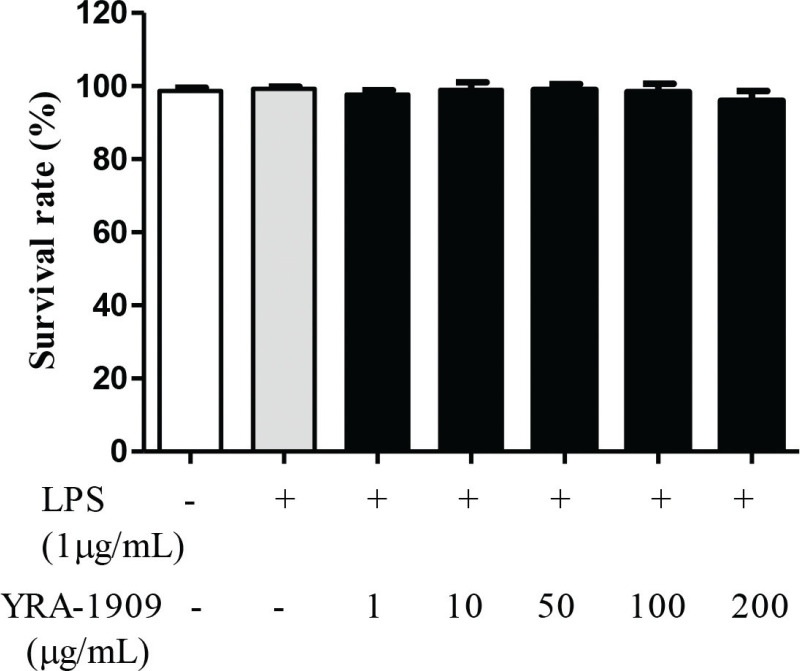
The effect of YRA-1909 on cell viability. Peritoneal macrophages were pretreated with 50–200 μg/mL YRA-1909, followed by LPS (1 μg/mL) for 18 h. Cytotoxicity was evaluated using the 3-[4,5-dimethylthiazol-2-yl]-2,5-diphenyltetrazolium bromide assay.

**Fig. 3:**
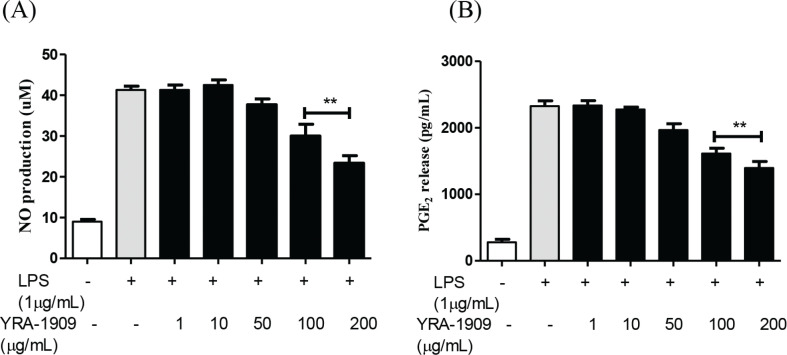
The effect of YRA-1909 on the production of inflammatory mediators (NO and PGE_2_) in LPS-induced rat peritoneal macrophages. (a) Cells were pretreated for 30 min with indicated concentrations of YRA-1909, followed by LPS (1 μg/mL) treatment and incubation for 18 h. (b) The conditions of sample treatment were identical to those described for Fig. 3a. Data represent mean ± SD. *P < 0.05 versus **P < 0.01 versus LPS-stimulated group.

To determine the suitable concentration range for investigating the effects of YRA-1909 on peritoneal macrophage viability, the cells were treated with YRA-1909 concentrations ranging from 50 to 200 mg/mL for 18 h and observed no significant changes in cell viability (Fig. 2). We, therefore, used YRA-1909 at these concentrations to investigate the effects of YRA-1909 on NO and PGE_2_ production in rat peritoneal macrophages. The potent macrophage activator LPS increased NO production relative to the control; however, this was inhibited by YRA-1909 in a dose-dependent manner (Fig. 3a). This effect was not due to cytotoxicity since viability was identical between YRA-1909-treated and control cells (Fig. 2). Similarly, LPS increased PGE_2_ production relative to the control group, while YRA-1909 abrogated this effect in a dose-dependent manner (Fig. 3b).

## Effect of YRA-1909 on LPS-induced proinflammatory cytokine production in rat peritoneal macrophages

**Fig. 4:**
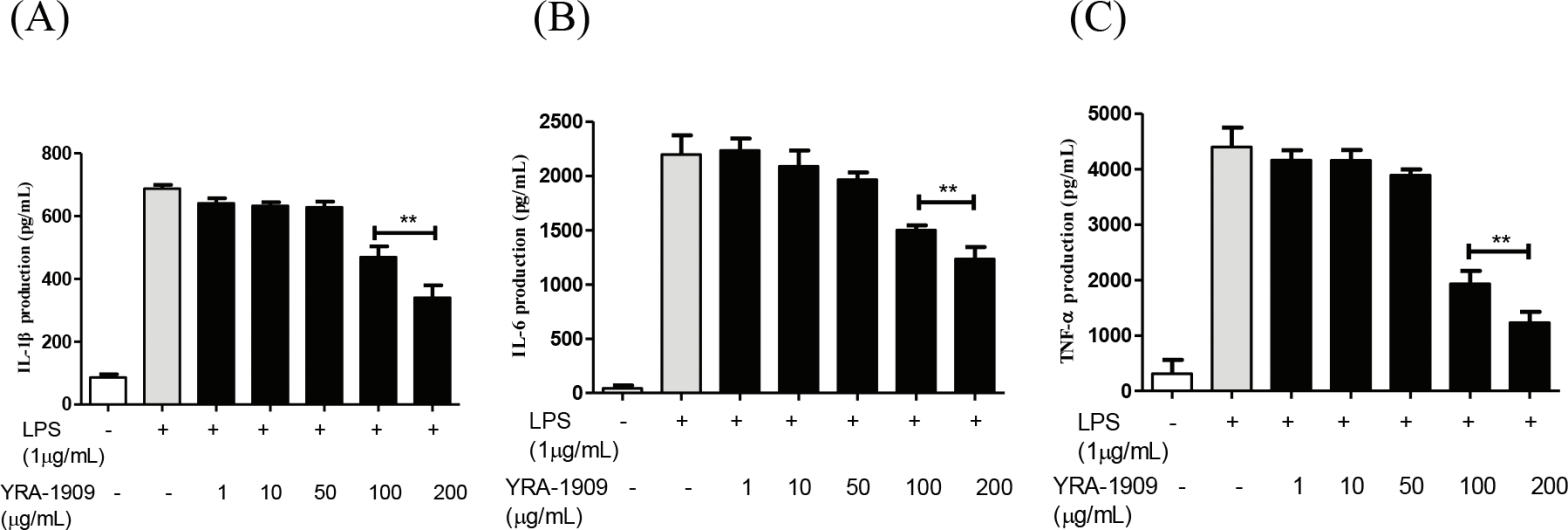
The effect of YRA-1909 on proinflammatory cytokine production in rat peritoneal macrophages. (a–c) The levels of IL-1β (a), IL-6 (b), and TNF-α (c) in the culture supernatant of cells stimulated with LPS (1 μg/mL) for 18 h in the presence of YRA-1909 (50, 100, and 200 μg/mL) were measured by ELISA. Data represent mean ± SD. *P < 0.05 versus **P < 0.01 versus LPS-stimulated group.

Several proinflammatory cytokines including IL-1β, IL-6, and TNF-α stimulate NO production in macrophages (37). LPS increased the levels of IL-1β, IL-6, and TNF-α in rat peritoneal macrophages, but this was reversed in a dose-dependent manner by YRA-1909 (Fig. 4).

## Effect of YRA-1909 on LPS-induced iNOS and COX-2 expression and COX-2 activity in rat peritoneal macrophagess

**Fig. 5:**
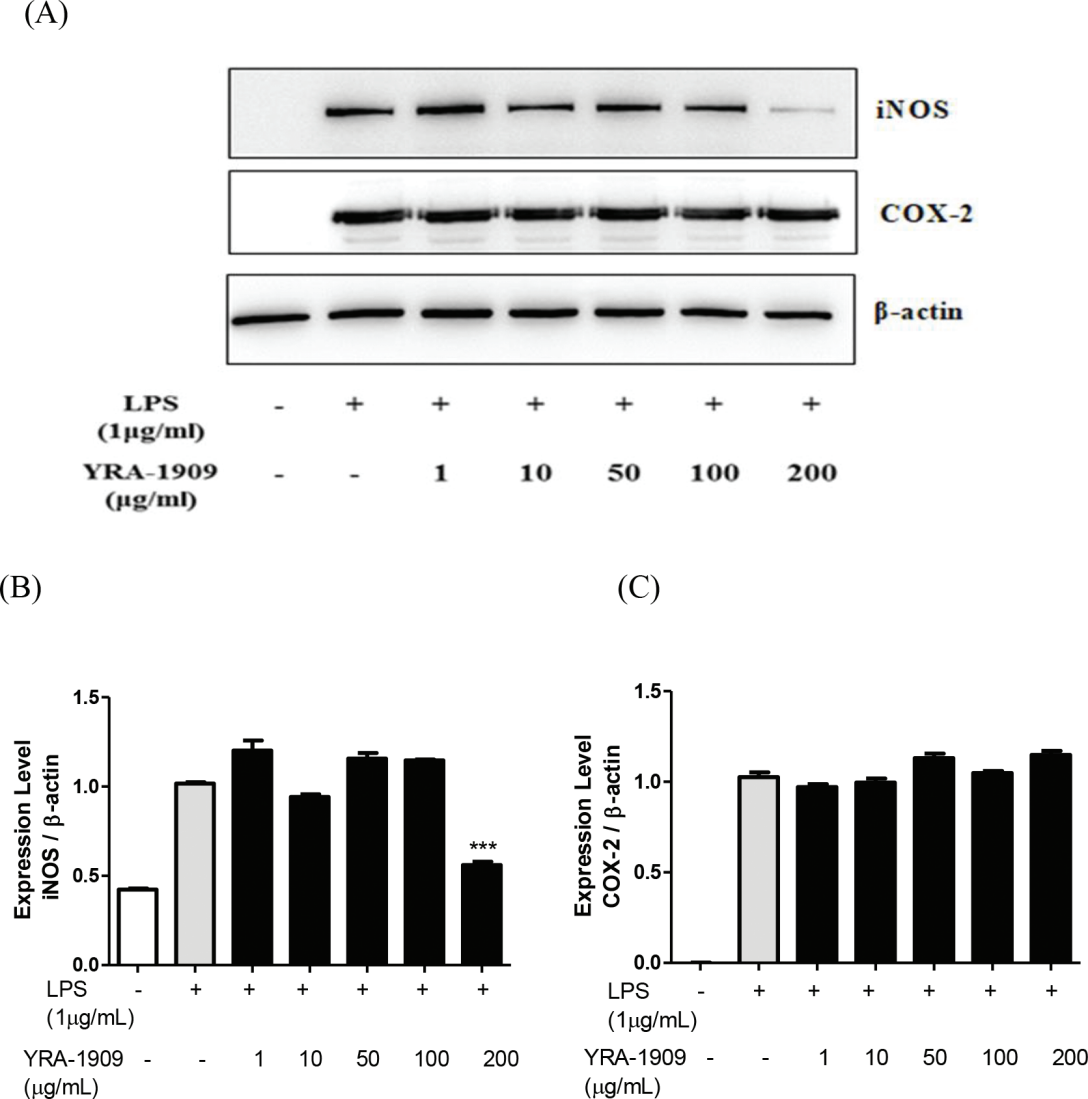
The effect of YRA-1909 on LPS-induced iNOS and COX-2 protein expressions in rat peritoneal macrophages. (a) Western blot analysis of iNOS and COX-2. (b) Densitometric analysis of iNOS relative to β-actin. (c) Densitometric analysis of COX-2 relative to β-actin. Cells were pretreated for 30 min with indicated concentrations of YRA-1909, followed by LPS (1 μg/mL) treatment and incubation for 18 h. iNOS and COX-2 protein levels were evaluated by western blotting.

We next investigated whether the inhibitory effects of YRA-1909 on the proinflammatory mediators NO and PGE_2_ are achieved through the modulation of iNOS and COX-2 expressions. The iNOS protein expression was increased in peritoneal macrophages treated with LPS for 18 h by western blotting, whereas YRA-1909 (50, 100, and 200 μg/mL) downregulated the iNOS expression relative to cells treated with LPS only; see Fig. 5. PGE_2_ is generated from arachidonic acid by the enzymatic action of COX during inflammation. LPS strongly stimulated the COX-2 protein expression, and this was unaffected by YRA-1909 at doses of 50, 100, and 200 μg/mL (Fig. 5).

**Fig. 6:**
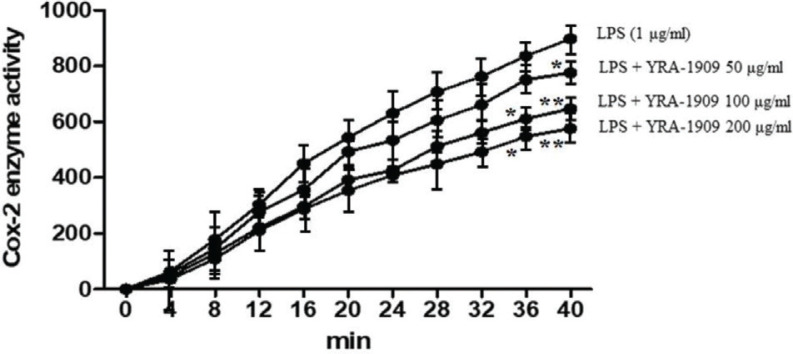
The effect of YRA-1909 on LPS-induced COX-2 activity in rat peritoneal macrophages. Cells were pretreated for 30 min with indicated concentrations of YRA-1909, followed by LPS (1 µg/mL) and incubation for 18 h. The COX-2 enzymatic activity was evaluated with a fluorescence assay. Data represent mean ± SD. *P < 0.05 versus **P < 0.01 versus LPS-stimulated group (analysis of variance followed by Dunnett’s test).

We examined the inhibitory activity of YRA-1909 on COX-2 and found that 50, 100, and 200 μg/mL YRA-1909 inhibited COX-2 activity in a dose-dependent manner. These results demonstrate that YRA-1909 suppresses the PGE_2_ production by decreasing the enzymatic activity rather than the expression of COX-2 (Fig. 6).

## Effect of YRA-1909 on NF-κB signaling

**Fig. 7:**
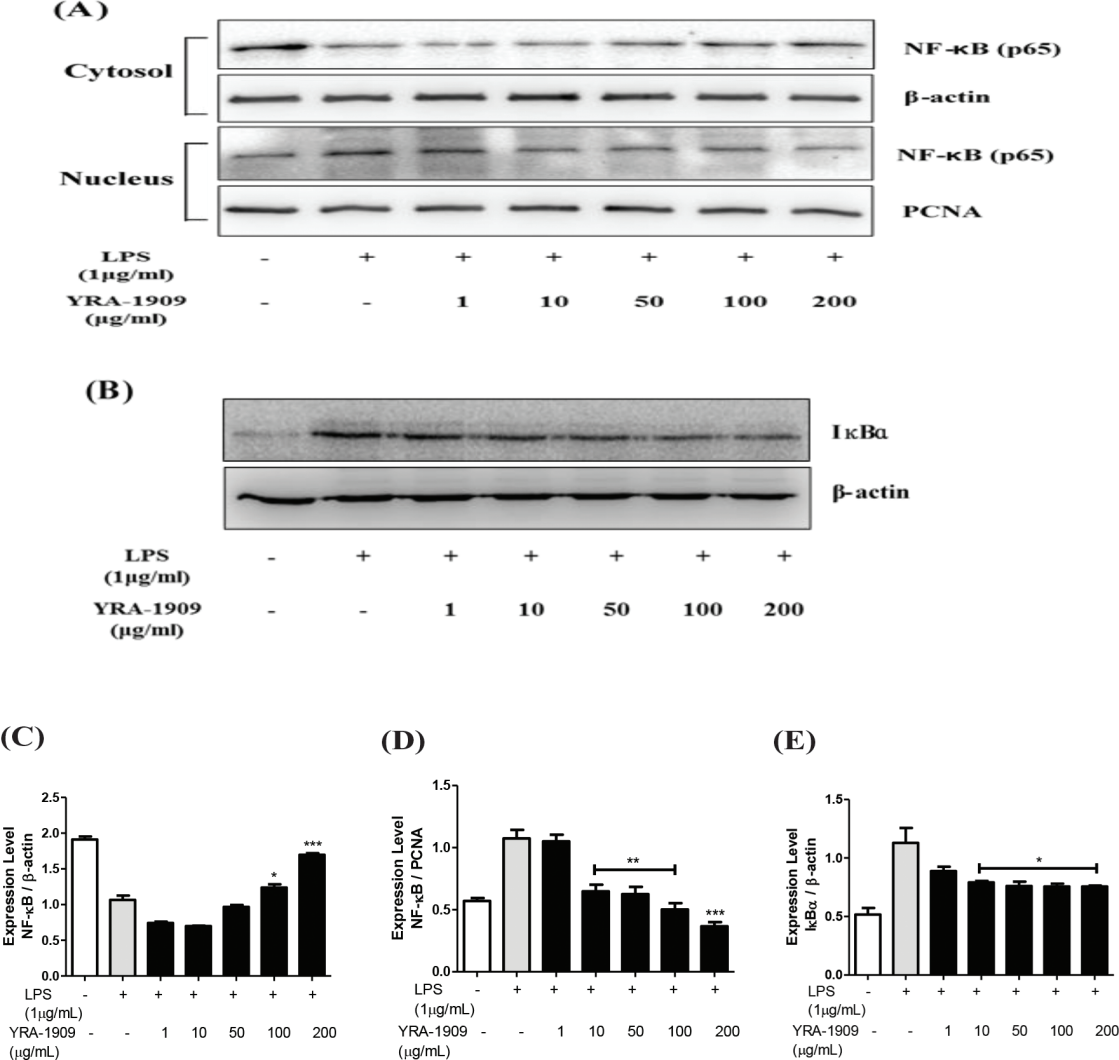
The effect of YRA-1909 extract on NF-κB translocation in rat peritoneal macrophages. (a) Western blot analysis of NF-κB. (b) Western blot analysis of Iκfα. (c) Densitometric analysis of NF-κB relative to β-actin. (d) Densitometric analysis of NF-κB relative to PCNA. (e) Densitometric analysis of IκBα relative to β-actin. Cells were pretreated with indicated concentrations of YRA-1909 for 1 h, followed by 1 µg/mL LPS for 1 h. Nuclear and cytosolic extracts were prepared, and equal amounts of proteins were resolved by western blotting. (a) NF-κB protein level was determined using an antibody specific for p65. (b) Samples were treated as described for Fig. 4a, and IκBα phosphorylation was analyzed by western blotting.

NF-κB is a transcription factor that regulates proinflammatory mediators such as COX-2, iNOS, IL-6, and TNF-α. YRA-1909 dose-dependently blocked the translocation of NF-κB (p65) from the cytosol to the nucleus as well as the phosphorylation of IκBα, which prevented its degradation (Fig. 7a, b). Thus, YRA-1909 reverses LPS-induced inflammation by modulating NF-κB signaling.

**Fig. 8:**
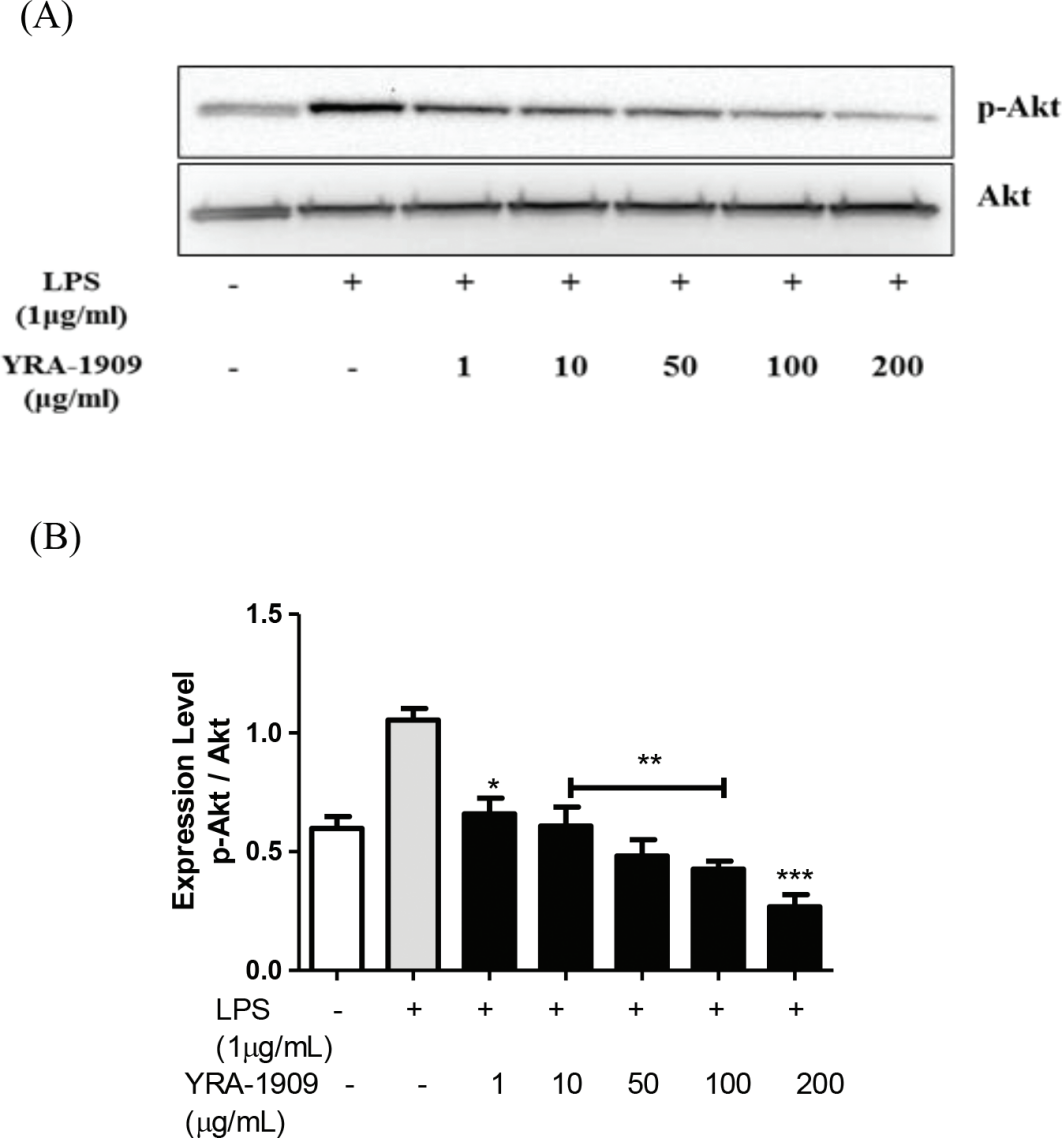
The effect of YRA-1909 on the activation of signaling pathways upstream of NF-κB. (a) Western blot analysis of p-Akt. (b) Densitometric analysis of p-Akt relative to Akt. Cells were pretreated with indicated concentrations of YRA-1909 and then stimulated with LPS (1 μg/mL). The activation Akt was evaluated by western blotting with phosphorylation-specific antibodies. *P < 0.05 versus **P < 0.01 versus ***P < 0.001 LPS-stimulated group.

To clarify the molecular mechanism underlying the anti-inflammatory effect of YRA-1909, we examined LPS-induced Akt activation in peritoneal macrophages. Cells were pretreated with YRA-1909 and then stimulated with LPS for different times. Preincubation with YRA-1909 prevented Akt phosphorylation, suggesting that the Akt pathway is involved in the YRA-1909-mediated inhibition of proinflammatory factors (Fig. 8).

## Effect of YRA-1909 on xylene-induced ear edema in mice

**Fig. 9:**
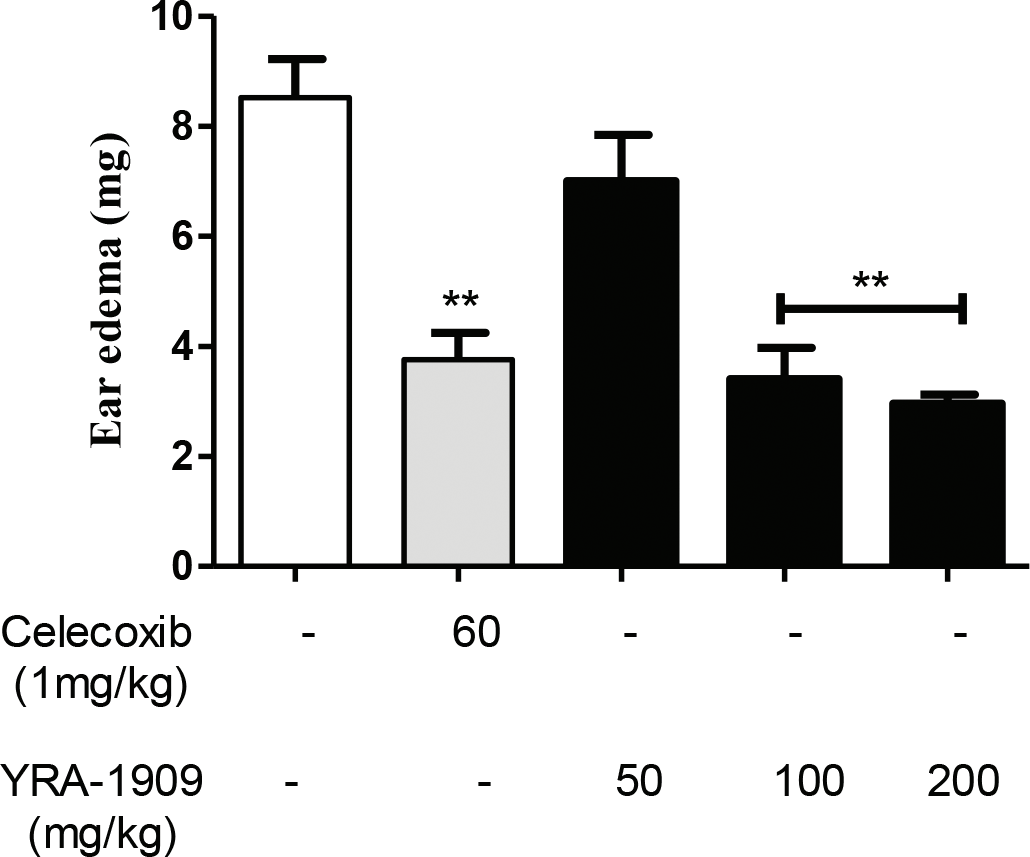
Effects of YRA-1909 on xylene-induced mouse ear edema. Both ears of each mouse were removed 1 h after 30 μL of xylene was applied to the right ear; the untreated left ear served as the control. Edema formation was calculated as described in the ‘Methods’ section. Data represent mean ± SD (*n* = 6). *P < 0.05 versus **P < 0.01 versus control.

The anti-inflammatory activity of YRA-1909 was evaluated in a mouse model of xylene-induced ear edema. The anti-inflammatory drug celecoxib administered at a dose of 60 mg/kg b.w. diminished xylene-induced ear edema by 63.3%, whereas 50, 100, and 200 mg/kg b.w. YRA-1909 dose-dependently reduced edema by 15.6, 43.0, and 54.4%, respectively (Fig. 9). Thus, YRA-1909 at the highest dose had an inhibitory effect comparable to that of the positive control celecoxib.

## Effects of YRA-1909 on carrageenan-induced paw edema in rats

Hind paw edema was induced in rats by intraplantar injection of carrageenan (Table 1). Pretreatment with YRA-1909 caused a dose-dependent reduction in hind paw edema. At 200 mg/kg b.w., YRA-1909 decreased edema formation by 36.1% at 5 h after treatment, as compared to the 55% reduction observed upon administration of 60 mg/kg b.w. celecoxib.

**Table 1:** The effect of YRA-1909 on carrageenan-induced rat paw edema over 5 h

Group	Dose (mg/kg)	Time (h)				
		1	2	3	4	5
Control	−	46.3 ± 2.1	68.9 ± 2.7	79.9 ± 1.5	85.2 ± 3.4	83.6 ± 1.4
Celecoxib	60	32.6 ± 1.2** (29.5)	38.5 ± 2.4** (44.1)	40.3 ± 2.3** (49.5)	41.1 ± 1.1** (51.8)	37.2 ± 1.9** (55.5)
	50	43.3 ± 1.9 (6.3)	61.5 ± 1.3** (10.7)	70.0 ± 1.2** (12.4)	75.2 ± 1.2** (11.7)	72.2 ± 1.3** (13.5)
YRA-1909	100	41.6 ± 1.9* (10.1)	58.2 ± 1.7** (15.5)	65.1 ± 1.5** (21.1)	69.3 ± 2.2** (22.9)	66.2 ± 1.0** (20.8)
	200	36.2 ± 1.7** (21.6)	50.2 ± 1.4** (27.2)	54.3 ± 1.9** (32.0)	56.3 ± 1.0** (33.9)	53.3 ± 1.2** (36.1)

Rat edema was induced in the right hind paw by the injection of 1% carrageenan following oral administration of YRA-1909. Saline (100 μL) was injected into the left paw as a control. Edema was measured at indicated time points after carrageenan injection using a plethysmometer. Data represent mean ± SD (*n* = 6).

* *P* < 0.05 versus

** *P* < 0.01 versus control.

## Effects of YRA-1909 on acetic acid-induced vascular permeability in mice

**Fig. 10:**
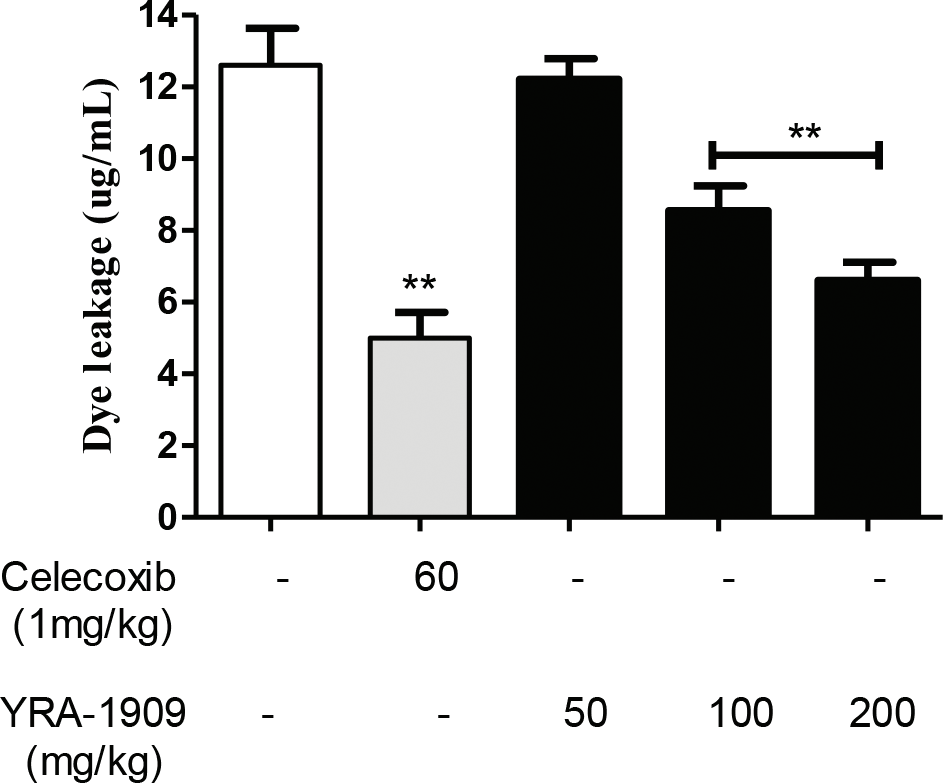
The effect of YRA-1909 on acetic acid-induced vascular permeability in mice. Mice were orally administered the indicated concentrations of YRA-1909 or celecoxib (positive control) before intraperitoneal injection of 0.6% acetic acid. Vascular permeability was evaluated based on dye leakage. Data represent mean ± SD (*n* = 6). *P < 0.05 versus **P < 0.01 versus control.

Celecoxib (60 mg/kg b.w.) inhibited acetic acid-induced dye extrusion into the peritoneal cavity by 61.6%; YRA-1909 at doses of 50, 100, and 200 mg/kg b.w. reduced dye extrusion in a dose-dependent manner by 11.7, 25.8, and 39.2%, respectively (Fig. 10).

## Effects of YRA-1909 on cotton pellet-induced granuloma formation in rats

We evaluated the effect of YRA-1909 on cotton pellet-induced granuloma formation and found that YRA-1909 at doses of 50, 100, and 200 mg/kg decreased granulomatous tissue formation by 6.9, 20.0, and 25.7%, respectively, as compared to the 38.2% reduction observed with 60 mg/kg b.w. celecoxib (Table 2).

**Table 2:** The effect of YRA-1909 on cotton pellet-induced granuloma in rats

Group	Dose (mg/kg)	Dry weight of granuloma (mg)	Inhibition (%)
Control	−	243.6 ± 16.5	−
Celecoxib	60	150.6 ± 11.0^**^	38.2
YRA-1909	50	226.8 ± 10.4	6.9
	100	195.0 ± 9.9^*^	20.0
	200	181.0 ± 5.1^**^	25.7

A 40-mg sterile cotton pellet was implanted into the groin region of rats, which were then intragastrically administered the indicated doses of YRA-1909 and celecoxib daily for 7 consecutive days. Granuloma formation was determined based on the dry weight of the pellet. Data represent mean ± SD (*n* = 6).

* *P* < 0.05 versus

** *P* < 0.01 versus control.

## Discussion

Recent studies have investigated the efficacy of plant-derived materials as an alternative to existing inflammatory agents, which can cause undesirable side effects (26). In this study, we investigated the biological properties of YRA-1909 and found that it was non-toxic and suppressed inflammation effects in both *in vitro* and *in vivo* models. This is the first report of the anti-inflammatory activity of YRA-1909.

We first confirmed that water extracts of *S. hexaphylla* leaves did not cause acute toxicity by treating rats intragastrically with 500, 1,000, and 2,000 mg/kg b.w. of the water extract daily for 2 weeks. We did not observe any abnormal behavior or mortality during the 14-day experiment and concluded that water extract of *S. hexaphylla* leaf is safe up to a dose of 2,000 mg/kg b.w. (not data shown).

Macrophages play an important role in diseases associated with excessive production of inflammatory mediators such as NO, PGE_2_, iNOS, and COX-2 and proinflammatory cytokines such as TNF-α, IL-1β, and IL-6 (38). In our study, YRA-1909 (100 and 200 μg/mL) reduced the NO production by inhibiting the iNOS expression. PGE_2_ is produced during inflammationby COX-2 (39). We found here that LPS-induced PGE_2_ production was attenuated by YRA-1909 at concentrations of 100 and 200 mg/kg. YRA-1909 also dose-dependently decreased the COX-2 enzymatic activity although the LPS-induced COX-2 protein expression was unaltered. Previous studies have shown that 8-hydroxydaidzein isolated from soybean fermented by *Aspergillus oryzae* suppressed the LPS-induced PGE_2_ production by modulatingCOX-2 activity without affecting its expression (9). Thus, YRA-1909 may act by directly blocking COX-2 enzymatic activity along with TNF-α, IL-6, and IL-1β expressions in LPS-activated rat peritoneal macrophages.

Inflammation involves the activation of many signaling pathways such as NF-κB, Akt, and mitogen-activated protein kinase in macrophages (40, 41). Some studies have shown that LPS can induce Akt phosphorylation and activate downstream NF-κB signaling (40, 42). Akt is a phosphorylation-activated kinase downstream of PI3K that plays an important role in cell death and survival (42). In its inactive form, NF-κB is present in the cytosol as a complex with its inhibitor IκBα. The activation of NF-κB in response to LPS stimulation leads to the degradation of IκBα and the release and nuclear translocation of NF-κB (41, 43). Our results demonstrate that YRA-1909 blocked the nuclear translocation of NF-κB by suppressing IκBα phosphorylation and degradation in LPS-treated rat peritoneal macrophages.

YRA-1909 showed potent anti-inflammatory effects in rodent models of acute and chronic inflammation. Ear edema is attributed to the release of inflammatory mediators such as histamine, serotonin, bradykinin, and prostaglandins that promote vasodilatation and increase vascular permeability (44).

Our findings indicate that YRA-1909 (100 and 200 mg/kg) may exert anti-inflammatory effects *in vivo* by preventing the release of these mediators. The carrageenan-induced paw edema model has been widely used to evaluate acute inflammation, which is divided into two phases (45): the early stage (1 h) involves the release of histamine and serotonin and the late phase (>1 h) is caused by the release of prostaglandin-like products (46, 47). YRA-1909 suppressed both phases of carrageenan-induced inflammation (1–5 h), similar to the effects of the standard anti-inflammatory drug celecoxib. Acetic acid-induced vascular permeability increases histamine, serotonin, bradykinin, and prostaglandin levels in the peritoneal fluid (44). YRA-1909 at concentrations of 100 and 200 mg/kg inhibited the acetic acid-induced increase in vascular permeability in mice. Cotton pellet-induced granuloma formation is a chronic inflammation model that is used to evaluate the transudative, exudative, and proliferative phases of inflammation (48). In the present study, YRA-1909 had a dose-dependent anti-inflammatory effect that was comparable to that of celecoxib. This is consistent with the reported anti-inflammatory effects of CGA, NCGA, and CCGA, the main bioactive compounds in YRA-1909 (30). Therefore, the screening of bioactive compounds should be conducted in future with a focus on the detailed mechanism.

## Conclusions

Our results demonstrate that YRA-1909 decreases the levels of proinflammatory mediators including iNOS, NO, COX-2, PGE_2_, and some cytokines in LPS-activated rat peritoneal macrophages by suppressing the Akt-dependent NF-κB activation. YRA-1909 also exerted the anti-inflammatory effects *in vivo*. Thus, YRA-1909 is a safe and effective alternative to NSAIDs and other conventional anti-inflammatory agents.

## Conflict of interest and funding

The authors declare no conflict of interest. This work was supported by the Support Program for Creative Industry Institutes (Commercial Bio-technology Sophistication Platform Construction Program, R0003950); and the Technology Innovation Program (10051145) funded by the Ministry of Trade, Industry & Energy (MOTIE, Korea). YRA-1909 has been registered at ClinicalTrials.gov (identifier: NCT03275025).

## Authors’ contributions

Jaeyong Kim, Hak-sung Lee, and Chulyung Choi conceived and designed the experiments, and prepared the manuscript; Huwan Kang, Gyuok Lee, Ji-seok Yoo, and Yongnam Lee performed the experiments and analyzed the data.
